# A Fast, Efficient and Easy to Implement Method to Purify Bacterial Pili From *Lacticaseibacillus rhamnosus* GG Based on Multimodal Chromatography

**DOI:** 10.3389/fmicb.2020.609880

**Published:** 2020-12-18

**Authors:** Raphael Dos Santos Morais, Sofiane El-Kirat-Chatel, Jennifer Burgain, Blandine Simard, Sarah Barrau, Cédric Paris, Frédéric Borges, Claire Gaiani

**Affiliations:** ^1^Laboratoire d’Ingénierie des Biomolécules, Université de Lorraine, Nancy, France; ^2^Laboratoire de Chimie Physique et Microbiologie pour les Matériaux et l’Environnement (LCPME), UMR 7564, CNRS-Université de Lorraine, Nancy, France; ^3^Institut Universitaire de France, Parris, France

**Keywords:** *Lacticaseibacillus rhamnosus* GG, SpaCBA pili, purification, multimodal chromatography, light scattering, atomic force microscopy

## Abstract

Pili are polymeric proteins located at the cell surface of bacteria. These filamentous proteins play a pivotal role in bacterial adhesion with the surrounding environment. They are found both in Gram-negative and Gram-positive bacteria but differ in their structural organization. Purifying these high molecular weight proteins is challenging and has certainly slowed down their characterization. Here, we propose a chromatography-based protocol, mainly relying on multimodal chromatography (core bead technology using Capto Core 700 resin), to purify sortase-dependent SpaCBA pili from the probiotic strain *Lacticaseibacillus rhamnosus* GG (LGG). Contrary to previously published methods, this purification protocol does not require specific antibodies nor complex laboratory equipment, including for the multimodal chromatography step, and provides high degree of protein purity. No other proteins were detectable by SDS-PAGE and the 260/280 nm ratio (∼0.6) of the UV spectrum confirmed the absence of any other co-purified macromolecules. One can obtain ∼50 μg of purified pili, starting from 1 L culture at OD_600nm_ ≈ 1, in 2–3 working days. This simple protocol could be useful to numerous laboratories to purify pili from LGG easily. Therefore, the present work should boost specific studies dedicated to LGG SpaCBA pili and the characterization of the interactions occurring with their protein partners at the molecular level. Moreover, this straightforward purification process might be extended to the purification of sortase-dependant pili from other Gram-positive bacteria.

## Introduction

Pili, or fimbriae, are length-variable proteinaceous appendages found at the surface of bacteria and archaea where they play a key role in interaction and/or adhesion with the surrounding environment (Berne et al., 2018). These proteins exhibit a high structural organization diversity (Proft and Baker, 2008). Some types of pili are found in both Gram-negative and Gram-positive bacteria and archaea, such as type IV pili (Hospenthal et al., 2017; Wang et al., 2019). Other pili are exclusive to Gram-negative bacteria, such as chaperone usher, and others are specific to Gram-positive bacteria (Hospenthal et al., 2017). Indeed, Gram-positive bacteria possess pili called sortase-dependent pili, named after the transmembrane enzyme catalyzing the reaction of polymerization. These pili are composed of three polymerized subunits, called pilin, covalently bound to each other, contrary to the other types of aforementioned pili. A single basal pilin allows for attachment to the cell wall, a major pilin is found in multiple copies which drives the length of the pilus, and a tip pilin located at the end of the pilus is responsible for interactions with the environment (Krishnan, 2015). Polymerization occurs through the reaction of a peculiar Lys of a pilin motif (exclusive to the basal and the major pilins) and a specific Thr found in the LPXTG motif which is common to the three pilins (Krishnan, 2015; von Ossowski, 2017). Remarkably, this kind of pili from *Corynebacterium diphtheriae* has successfully been rebuilt *in vitro* (Chang et al., 2018) and the reaction occurring between the pilin and the LPXTG motifs was exploited for protein labeling (McConnell et al., 2018). Sortase-dependent pili were thought to be exclusive to pathogens, but in the late 2000s, they were observed by transmission electron microscopy (Kankainen et al., 2009) on the non-pathogenic probiotic bacterium *Lactobacillus rhamnosus GG* (LGG) (Capurso, 2019), which was recently reclassified as *Lacticaseibacillus rhamnosus* GG (Zheng et al., 2020). When overexpressed, SpaCBA pili have also been detected on a *Lactococcus lactis* strain (Tarazanova et al., 2016). LGG SpaCBA pili can reach up to 1 μm in length and 10 to 50 copies are found per cell (Kankainen et al., 2009). They are composed of a basal pilin (SpaB, ∼20 kDa), a major pilin (SpaA, ∼30 kDa) and a tip pilin (SpaC, ∼90 kDa), whose genes are clustered (Kankainen et al., 2009). The estimated molecular weight of a single pilus is 1 to 3 MDa, depending on its length. These pilins likely self-organize according to a the classical way described above to form a functional pilus (von Ossowski, 2017; Kant et al., 2020). A model of LGG pilus where SpaC is found all along the pilus was proposed following immunogold labeling and visualization of pilus structure by electron microscopy (Reunanen et al., 2012). This hypothetical model might be erroneous since polyclonal antibodies might have recognized structural motifs shared by the pilins (von Ossowski, 2017), i.e., the Ig-like fold made of seven β-sheets, of the CnaB domains found in the three pilins. A recent study appears to remove this ambiguity since when monoclonal antibodies were used, SpaC was only found at the tip of the pilus according to immunogold labeling in transmission electron microscopy experiments (Kant et al., 2020). Moreover, SpaCBA pili are thought to be glycosylated through SpaC, with fucose and mannose residues (Tytgat et al., 2016). Nonetheless, SpaC harbors a structural homology with a lectin specific to mannose residues and might also contain some properties specific to fucose binding lectin (Kant et al., 2020). Therefore, the actual glycosylation of SpaC and/or its lectin capacity still need further investigation. Interestingly, chemically-induced derivatives of LGG (non-GMO) have been generated in order to improve adhesion and the associated putative probiotic role through SpaCBA pili (Rasinkangas et al., 2020).

Most studies done on LGG have aimed to decipher the interaction with molecules found in the gastro-intestinal tract (von Ossowski et al., 2010; Lebeer et al., 2012), where bacteria can exert their probiotic action. These properties include preventing pathogen adhesion, producing antimicrobial compounds (e.g., bacteriocins), fighting for nutrients, improving the epithelial barrier through mucin production, and modulating the immune response (Suez et al., 2019). Over the last decade, the use of probiotics has attracted increasing interest among consumers, whether consumed within food or as a dietary supplement. This keen interest has increased incessantly, with the ensuing pros and cons (Suez et al., 2019). One major concern when used as probiotic is to determine whether pili remain intact after industrial processes including spray-drying and the subsequent storage (Agudelo et al., 2017; Broeckx et al., 2017; Guerin et al., 2017; Gomand et al., 2019; Kiekens et al., 2019). One solution to protect bacterial pili during industrial processes consists to of encapsulating them in a dairy product-based matrix including whey proteins. This has given rise to recent studies focusing on the interaction of LGG with dairy components (Guerin et al., 2018a,b; Dos Santos Morais et al., 2020). It was demonstrated that SpaCBA pilus is capable of establishing interactions with β-lactoglobulin, while no interactions have been detected with other whey proteins (BSA and α-lactalbumin) or with caseins (Burgain et al., 2014; Guerin et al., 2016). A divide-and-conquer approach is often employed to determine the role of each pilin subunit requiring the production of recombinant proteins (von Ossowski et al., 2010). *Via* this approach, structural characterizations of SpaCBA pilus subunits though X-ray crystallography (XRC) aiming to determine the structural organization are ongoing. Indeed, the XRC structure of SpaA has been made available (Chaurasia et al., 2016), such as that of SpaC (Kant et al., 2020), and efforts are currently being made to solve the structure of SpaB (Kumar Megta et al., 2019). For a better characterization of the pili function, it is essential to obtain them in a native state to take into account the potential effect of adjacent pilins, whatever the context of the gastro-intestinal tract molecules or food components.

Only few publications report the purification of native SpaCBA pili from LGG and it sometimes requires the use of specific antibodies that are non-commercially available (Reunanen et al., 2012). Others describe a centrifuge-based simple protocol to purify these pili (Tripathi et al., 2012), but exopolysaccharides (EPS) were likely co-purified during this procedure. Although the above protocol was improved by adding a size exclusion chromatography step, the required equipment is specific and costly (Tytgat et al., 2016). In the present study, an alternative protocol mainly based on multimodal chromatography (MMC, Capto Core 700) was developed. This technology combines hydrophobic interaction, ion exchange and size exclusion chromatographies, and was initially used to purify viruses (Blom et al., 2014), but it can be extended to very large molecules including pili from LGG. Furthermore, this method is straightforward and requires minimal laboratory equipment. Once purified, pili were characterized by mass spectrometry, size exclusion chromatography coupled to triple detection array (SEC-TDA) and successfully imaged by atomic force microscopy (AFM).

## Materials and Methods

### Bacterial Culture

#### Bacterial Strains and Pre-culture

The model strain *Lacticaseibacillus rhamnosus* GG (ATCC53103) (LGG WT) and pili defective derivative mutant LGG Δ*spaCBA*:Tc^*R*^ (CMPG 5357, LGG Δ*spaCBA*) (Lebeer et al., 2012) were used in this study. A bacterial pre-culture was prepared by adding 500 μL of bacterial glycerol stock (stored at −80°C) into 50 mL (∼1/100^*e*^) of MRS medium (Biokar) and placed overnight at 37°C without agitation.

#### Static Culture

*Lacticaseibacillus rhamnosus* GG WT or LGG Δ*spaCBA* culture was prepared by seeding approximately 1 L of fresh MRS with the pre-culture (∼1/50^*e*^) and was incubated at 37°C without agitation until the OD_600nm_ reached ∼1–1.2 (4–5 h). Bacteria were harvested by centrifugation (3000 × *g*, 10 min, 20°C). The pellets were washed with PBS pH 6.8 (Sigma) and the suspended cells were centrifuged again (3000 × *g*, 10 min, 20°C).

#### Bioreactor Culture

To increase the biomass and thereby the quantity of pili, LGG WT was grown in a bioreactor under controlled pH (6.8, adjusted with 2 M NaOH) and temperature (37°C) under gentle homogenization. Five liters of MRS were seeded with 150 mL of pre-culture (cf. section “Bacterial Strains and Pre-culture”). Bacterial growth was monitored by measuring OD_600nm_ every hour and by monitoring NaOH consumption. Cells were harvested by centrifugation (3000 × *g*, 10 min, 20°C) in the late exponential phase when OD_600nm_∼ 6–7 (∼5 h). They were washed with PBS pH 6.8, then centrifuged again (3000 × *g*, 10 min, 20°C).

### Protein Purification

#### Cell-Wall Digestion

**FIGURE 1:**
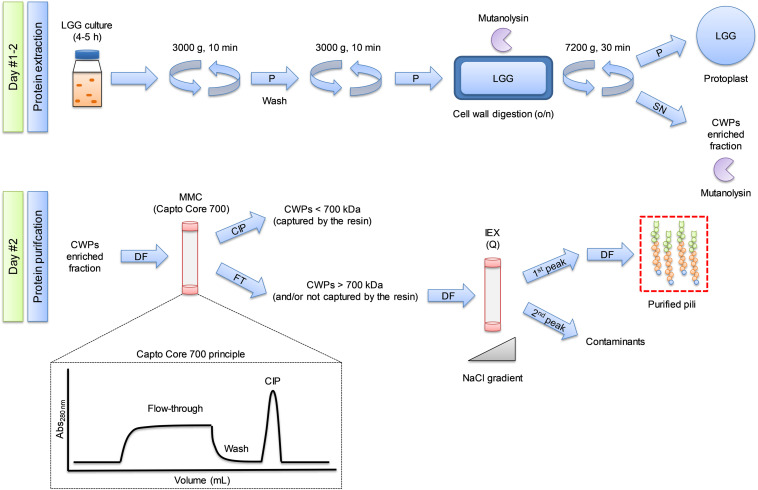
Overview of the purification process of bacterial SpaCBA pili from Lacticaseibacillus rhamnosus GG (LGG). Protocol developed in the present study relying mainly on multimodal chromatography and the core bead technology using Capto Core 700 resin. SN: supernatant, P: pellet, DF: diafiltration, CIP: cleaning in place. FT: flow-through, CWP: cell wall protein, MMC: multi modal chromatography, IEX: ion exchange chromatography.

For clarity, Figure 1 shows a diagram summarizing the extraction and purification protocols. Washed pellets were suspended in digestion buffer (50 mM Tris pH 6.8, 150 mM NaCl, 2 mM MgCl_2_, 20% sucrose) supplemented with mutanolysin (50–100 U/mL). The cell wall was digested overnight at 37°C under gentle agitation to avoid cell lysis. The measured pH after enzymatic digestion was 4.2 and was adjusted to 6.8 with 500 mM Tris pH 6.8. The suspension was supplemented with 5 U/mL of DENARASE (C-LEcta) to digest potentially released nucleic acid material in case of eventual cell lysis, and centrifuged (7200 × *g*, 30 min, 20°C). The supernatant, containing SpaCBA pili, was carefully collected, diluted in 25 mM Tris pH 6.8, and filtered through 0.45 μm PES (Polyethersulphone) filter.

#### Diafiltration

All diafiltration steps were performed with Amicon ultra-15 concentrators (100 MWCO, Millipore) or Amicon stirred-cell (200 mL, 100 MWCO, Millipore) under N_2_ pressure (2.5 bar) to handle larger volumes. Buffer exchanges differed according to the following purification step.

#### Chromatography

All subsequent chromatography steps were performed with an ÄKTA Start liquid chromatography system (GE Healthcare).

##### Multimodal chromatography (MMC)

The clarified solution was loaded at 2 mL/min on Capto Core 700 columns (GE Healthcare) mounted in series (5 × 1 mL HiTrap column and 1 × 4.7 mL HiScreen column) equilibrated with 25 mM Tris pH 6.8. The flow-through was saved, then the columns were washed with the same buffer until the Abs_280nm_ returned to the initial baseline. The columns were regenerated with 1M NaOH, 30% isopropanol (cleaning in place or CIP) to desorb captured molecules.

##### Size Exclusion Chromatography (SEC)

Concentrated fractions (3–5 mL) from the flow-through of the MMC step were filtered through a 0.22 μm PES filter prior to injection onto a Sephacryl HR 400 column (void volume ∼40 mL, total volume ∼120 mL, GE Healthcare) equilibrated with PBS pH 6.8 at 0.8 mL/min.

##### Ion Exchange Chromatography (IEX)

The diafiltered solution from MMC was injected onto strong anion exchange columns (2 × 5 mL HiTrap Q HP, GE Healthcare) at 5 mL/min, previously equilibrated with 25 mM Tris pH 6.8 (buffer A). Then the columns were washed with buffer A until the Abs_280nm_ returned to the initial baseline. Captured molecules were eluted with a linear gradient at 2 mL/min reaching 100% 25 mM Tris pH 6.8, 1M NaCl (buffer B) in 50 mL.

### SDS-PAGE Analysis and Protein Quantification

Aliquots of each step of purification were collected and acetone-precipitated if required. Purification steps were analyzed by SDS-PAGE (precast 4–15% or 4–20% gradient gel, Bio-Rad) in reducing condition (50 mM dithiothreitol, DTT) and stained with Coomassie Blue (Instant Blue, Expedeon). Protein concentration was estimated spectrometrically (NanoDrop 2000c, Thermo Scientific) considering the SpaA molar extinction coefficient (39,880 M^–1^.cm^–1^) and MW (29.2 kDa) without the signal peptide and the C-term part after the ^301^LPXT^304^G motif (^34^DTN…LPHT^304^). Protein purity toward other types of macromolecules was estimated considering the 260/280 ratio and visual inspection of the UV-spectrum.

### Mass Spectrometry

Two different setups were employed in this study: LC-MALDI and LC-ESI.

#### LC-MALDI

Gel bands from SDS-PAGE analysis were processed by successive washes at 20°C under agitation in a 50 μL volume. For cysteine reduction and alkylation, bands were incubated in 100 mM ammonium bicarbonate (AB), then in AB containing 50 mM DTT for 45 min, washed once in AB, and finally incubated in AB containing 50 mM IAA (iodoacetamide) for 45 min. Bands were washed through two cycles as follows: 15 min in AB/ACN (acetonitrile), 1:1, 15 min in AB. Finally, they were dehydrated twice in ACN and dried in a vacuum concentrator for one hour. Bands were digested with 50 ng trypsin (sequencing grade, Promega) overnight in 7 μL AB/water, 1/1. The next day, the peptides were extracted twice in 10 μL ACN, 80%, TFA 1% for 7 min under sonication. This procedure was done twice. Peptide extracts were pooled and dried in a vacuum concentrator, resuspended in 10 μl 2% ACN, 0.1% TFA and processed for fractionation by nano HPLC.

Nano HPLC was carried out with a UltiMate 3000 system (Thermo Scientific) equipped with a 20 μL sample loop, a pepMap 100 C18 desalting precolumn (Dionex) and a 15 cm pepMap RSLC C18 fractionation column (Dionex). Samples (5 μL) were injected using the μL pickup mode and eluted by a 2 to 45% ACN gradient over 30 min at 300 nl/min. Fractions (170, 9 s each) were collected on a ProteineerFcII (Bruker) over 25.5 min and eluates were directly mixed on MTP-1536 TF target (Bruker) spots to α-cyano-4-hydroxycinnamic acid (Bruker). LC-MALDI runs were processed using dedicated automatic methods piloted by WARP-LC software on an Autoflex speed MALDI-TOF/TOF mass spectrometer (Bruker) in the 800–3,500 mass range, using next-neighbor external calibration for all MALDI spots, using 2000 random laser shots per spot at a 2000 Hz frequency. Masses detected with S/N above 20 were selected for TOF/TOF fragmentation in LIFT mode.

Peptide assignments were performed from TOF/TOF spectra by Mascot Server interrogation (v2.4.1, Matrix Science) piloted and compiled by Proteinscape. The database search parameters were as follows: mass tolerance for precursors = 50 ppm; mass tolerance for fragments = 0.8 Da; enzyme = trypsin with one missed cleavage allowed; protein modifications = carbamidomethylation of cysteines (variable) and oxidation of methionines (variable). The specific database was [Uniprot proteome UP000000955, 2019/06/28, 2877 sequences, (Kankainen et al., 2009)]. Proteins were considered as identified when at least two peptides passed the Mascot score with *p* < 0.05 threshold.

#### LC-ESI

Gel pieces from SDS-PAGE were excised and cut into small cubes and processed with the in-gel digestion kit (Thermo Scientific). Briefly, the gels pieces were destained with acetonitrile 30%, reduced with TCEP [tris(2-carboxyethyl)phosphine], alkylated with IAA and digested with trypsin overnight at 37°C. The peptides were extracted according to a previously published method (Lavigne et al., 2012).

Peptides were analyzed on a Vanquish quaternary UHPLC system (Thermo Scientific) in-line with a photodiode array detector (PDA) and an Orbitrap ID-X Tribrid mass spectrometer (Thermo Scientific) equipped with an atmospheric pressure ionization interface operating in electrospray mode (ESI). Sixteen microliters of peptides were separated on an Acclaim 120 C18 column (100 mm × 2.1 mm–2.2 μm, Thermo Scientific) maintained at 30°C. The flow rate was set at 200 μl/min and mobile phases consisted in water modified with formic acid (0.1%) for A and acetonitrile modified with formic acid (0.1%) for B. Peptides were eluted using a gradient step of 5 to 95% B for 40 min, then an isocratic step was applied at 95% B for 10 min to wash the column, before returning to the initial composition of 5% B for 5 min to realize the equilibrium. Mass analysis was carried out in ESI positive ion mode (ESI +) and mass spectrometry conditions were as follows: spray voltage was set at 3.5 kV; source gases were set (in arbitrary units/min) for sheath gas, auxiliary gas and sweep gas at 35, 7, and 0, respectively; vaporizer temperature and ion transfer tube temperature were both set at 300°C. Survey scans of peptide precursors from 150 to 2,000 m/z were performed at 60 K resolution (full width of the peak at its half maximum, fwhm, at 200 m/z) with MS parameters as follows: RF-lens, 35%; maximum injection time, 50 ms; data type, profile; internal mass calibration EASY-IC TM activated; custom AGC target; normalized AGC target: 25%. A top speed (0.6 s) data-dependent MS2 was performed by isolation at 1.5 Th with the quadrupole, HCD fragmentation with a stepped collision energy (25, 35, and 50) and MS analysis in the Orbitrap at 15 K resolution (high resolution MS/MS analysis). Only the precursors with intensities above the threshold of 2.104 were sampled for MS2. The dynamic exclusion duration was set to 2.5 s with a 10 ppm tolerance around the selected precursor (isotopes excluded). Other MS2 parameters were as follows: data type, profile; custom AGC target; normalized AGC target: 20%. Mass spectrometer calibration was performed using the Pierce FlexMix calibration solution (Thermo Scientific). MS data acquisition was carried out utilizing the Xcalibur v. 3.0 software (Thermo Scientific).

LC-MSMS data were processed and analyzed with Mascot Distiller (v2.7, Matrix Science) and Mascot Server (v2.7, Matrix Science). The database search parameters were as follows: mass tolerance for precursors = 10 ppm; mass tolerance for fragments = 20 ppm; enzyme = trypsin with two missed cleavages allowed; protein modifications = carbamidomethylation of cysteines (fixed) and oxidation of methionines (variable). The databases were [#1: Contaminants 2016/01/29, 247 sequences, #2: SwissProt 2019/11, 561 568 sequences, and #3: Uniprot_LGG_UP000000955, 2020/02/25, 2877 sequences, (Kankainen et al., 2009)]. Proteins were considered as identified when at least two peptides passed the Mascot score with *p* < 0.01 threshold scores.

#### Dynamic Light Scattering (DLS)

The polydispersity and the hydrodynamic radius (*R*_*h*_) of the pili were estimated by DLS using a Zetasizer instrument (Nano ZS, Malvern Panalytical). The sample was filtered through a 0.22 μm PES-filter just before analysis. Measurements were performed at 20°C in PBS buffer (pH 7.4) using a quartz cell. Data were processed with the Zetasizer software (v7.13, Malvern Panalytical) with default parameters. The size distribution by intensity was obtained from 6 successive experiments to ensure sample stability.

### Size-Exclusion Chromatography Coupled to Triple Detection Array (SEC-TDA)

Size exclusion chromatography experiments were performed with a HPLC pump (LC10AD, Shimadzu) coupled to an autosampler (Viscotek VE 2001, Malvern Panalytical) and a multi-detector system recording light scattering (RALS/LALS) intrinsic viscosity and refractive index signals (Viscotek TDA305, Malvern Panalytical). The HPLC SEC column (BioSec5 500 Å, 5 μm, 7.8 mm ID × 300 mm, void volume ∼6 mL, total volume ∼ 12.5 mL, Agilent) was equipped with a post-column nylon filter (0.22 μm). The column was equilibrated with PBS (pH 6.8) supplemented with sodium azide 0.02%. The flow rate and the temperature were 0.35 mL/min and 30°C respectively. Data were processed with the OmniSEC software (v5.12, Malvern Panalytical). The calibration procedure was done with BSA (Sigma) and cross-validation was performed with β-lactoglobulin (Sigma). The refractometer was used as the concentration detector and the refractive index increment value (*dn/dc*) used to determine the molecular weight was 0.185 mL/g. Samples were diafiltered in the aforementioned buffer and filtered through a 0.22 μm PES-filter just before injection.

### Atomic Force Microscopy (AFM) Imaging

Two hundred microliters of LGG WT cell suspension cultivated as in section “Bacterial Strains and Pre-Culture” in MRS or two hundred microliters of purified pili (diluted at 50 and 2.5 mg/L) in 25 mM Tris pH 6.8, 150 mM NaCl were deposited on freshly cleaved mica substrates and allowed to settle for 2 h. Then the surfaces were gently rinsed by immersion in 3 baths of ultrapure water and dried overnight at 30°C before imaging. Images were obtained in peak force tapping mode with a Bioscope Resolve AFM (Bruker corporation, Santa Barbara, CA, United States), using SNL cantilevers (Bruker corporation, nominal spring constant of ∼0.24 N.m^–1^) and with a maximum applied force of 5 nN.

## Results and Discussion

### Purification Protocols Available in the Literature

The literature was investigated to determine available protocols to purify pili from LGG. First, (Tripathi et al., 2012) developed a simple centrifuged-based protocol. The bacterial culture was subjected to two successive centrifugation steps, one at 8000 × *g* to remove pili from bacteria due to shear stress and a second one at 20000 *g* to pellet them. The authors characterized the pili by AFM and admitted that exopolysaccharides (EPS) could have co-precipitate during the 20000 × *g* centrifuge step, meaning that EPS contaminants could interfere with other techniques. Moreover, other cell wall proteins (CWPs) could have been released during the centrifugation steps and probably altered the pili purity even if they were not visible by AFM, due to their relative small sizes.

In the study by Tytgat et al. (2016) LGG was grown in an industrial whey permeate, as previously done (Laakso et al., 2011), and cells were concentrated by microfiltration. Cells were then centrifuged at 20000 × *g*, and the authors stated that the pili were found in the supernatant instead of the pellet, contrary to Tripathi et al. (2012). Then they added a second step of purification consisting of a size exclusion chromatography step. Pili were presumably found in the void volume of the column. In this case, EPS might have co-eluted with the pili during this step and SEC requires specific equipment.

Instead of simply centrifuging bacterial cells, (Reunanen et al., 2012) digested the cell-wall with mutanolysin and lysozyme in order to release CWPs including pili. Then CWPs were injected onto a column functionalized with polyclonal antibodies anti-SpaCBA, allowing them to remove contaminants. It is worth noting that to destabilize the specific antibody-antigen bound, it is necessary to elute the bound proteins at low pH (∼ 3). This could damage the native state of the pili even if pH was neutralized directly in collection tube. Although efficient, this protocol requires the use of specific antibodies that are not commercially available. Moreover, the intermediates of polymerization were still present in the purified fractions, as shown by western blots.

### Extraction of SpaCBA Pili *via* Enzymatic Digestion

In order to purify the pili, the simplest protocol available (i.e., the centrifuge-based protocol) developed by Tripathi et al. (2012) and improved by Tytgat et al. (2016) was tested. Unfortunately, for us it was not possible to extract any detectable CWPs in the supernatant or in the pellet of each centrifuge steps based on Coomassie blue staining after SDS-PAGE analysis. The same result was obtained after resuspending the cells vigorously with a vortex during 1 min before the 8000 × *g* centrifuge step (Supplementary Figure 1A).

As a second option, it was decided to employ a double enzymatic digestion of the CW with mutanolysin and lysozyme, as done by Reunanen et al. (2012) (Supplementary Figure 1B). Mutanolysin being quite expensive, the enzyme concentration was lowered to 50–100 U/mL and the digestion time increased from 2 h to overnight (∼12 h) under gentle agitation to avoid protoplast lysis. Later, only mutanolysin was employed as it was not necessary to use lysozyme to obtain a satisfactory CW digestion (data not shown). As expected, the enzymatic digestion of LGG CW released CWPs, as previously done to purify pili from *Streptococcus pneumoniae* (Hilleringmann et al., 2008).

**FIGURE 2:**
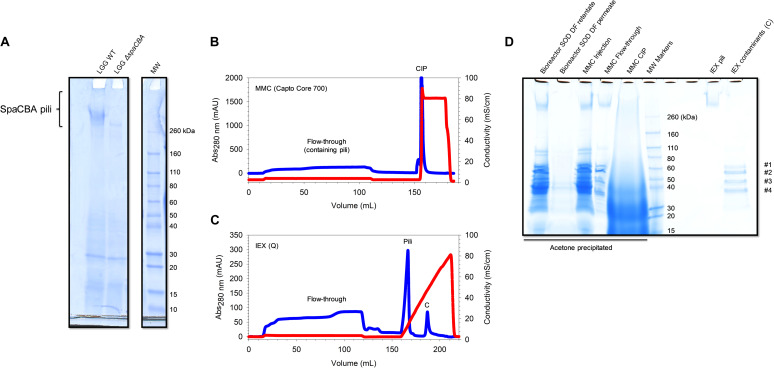
SpaCBA pili purification from LGG. **(A)** SDS-PAGE analysis highlighting the absence of pili production in LGG Δ*spaCBA* **(B)** Multimodal chromatogram (Capto Core 700) and **(C)** ion exchange chromatogram (HiTrap Q HP). Blue lines correspond to Abs_280nm_ signal and red lines to conductivity. **(D)** SDS-PAGE analysis throughout the purification process. SOD: supernatant of digestion, DF: diafiltration, MMC: multimodal chromatography, CIP: cleaning in place, IEX: ion exchange chromatography.

For the development of the protocol of purification, LGG WT was cultivated in MRS medium in static condition under uncontrolled pH, and LGG Δ*spaCBA* was used as a negative control. The difference in migration in SDS-PAGE, only visible at the top of the gel, clearly indicated the absence of pili on LGG Δ*spaCBA* (Figure 2A). This approach would allow for a rapid identification of pili at the protein level for other lactic acid bacteria. Currently, the presence/absence of pili is limited to genome mining and analysis, without any guarantee of gene expression and protein production. To increase the biomass and the quantity of pili, LGG WT was grown in a bioreactor under controlled pH (6.8) and gentle homogenization. For both static and bioreactor cultures, cells were harvested by centrifugation in the late-exponential growth phase where pili are still expressed (Laakso et al., 2011).

### Purification of SpaCBA Pili *via* Chromatography

Having a CWP-enriched fraction, the chosen approach to separate pili from other protein contaminants was ratiocinated based on their size. Considering the length of SpaA (∼9.3 nm) (Chaurasia et al., 2016), and taking into account the fact that the size of pili ranges from ∼300 nm to up to 1 μm, it was roughly estimated that a pilus is composed of ∼33–100 SpaA subunits. The MW of SpaA being ∼30 kDa, it can be reasonably assumed that a single pilus has a MW of 1–3 MDa. With this information in mind, the use of multimodal chromatography (MMC, Capto Core 700) to purify the pili was chosen. This core bead technology was initially developed for virus purification (Blom et al., 2014), but should be adapted for the purification of high molecular weight (HMW) proteins including bacterial pili (Figure 1). This technique is a flow-through-based method. Molecules having a MW > to 700 kDa cannot enter the beads while molecules having MW < to 700 kDa should enter the beads and be captured *via* electrostatic and/or hydrophobic interactions *via* the octyl amine ligand that is positively charged at neutral pH. Positively charged molecules that are not retained by the hydrophobic part of the ligand will also go through the beads. Up to 10 column volume (CV) can be loaded onto the column, much more than the 0.5–4% CV of sample volume that is recommended to be loaded on SEC columns. This unequivocally increases the amount of samples as well as the rapidity of the purification. Notably, the technology is available for 400 kDa molecules (Capto Core 400).

The supernatant of digestion (SOD) was injected on Capto Core 700 columns and the flow-through was saved. The column was washed with PBS and regenerated with NaOH/isopropanol mixture to desorb simultaneously molecules bound *via* hydrophobic and/or electrostatic interactions (Figure 2B). Following SDS-PAGE analysis (Figure 2D), one smeared band in the HMW part on the gel (>260 kDa) was observed and other bands from 30 to 60 kDa were noticed in the flow-through fraction. In the cleaning-in-place fraction, a smear was noted down to 10 kDa, meaning that plenty of CWPs were successfully bound to Capto Core 700 resin. Importantly, it was noted that running SDS-PAGE at low current (∼100V) improved the resolution at HMW and gave a smeared band as expected, corresponding to pili with different but close sizes. Interestingly, the major pilin SpaA harbors two isopeptide bonds between K47 and N172 and K184 and D295, one in each CnaB domain (Chaurasia et al., 2016). If those bonds are maintained during SDS-PAGE analysis, it could drastically decrease the apparent MW of the pili since the proteins might not be fully unfolded by SDS. Indeed, CnaB domains are predicted to be inextensible, contrary to CnaA domains, due to the difference in position of the isopeptide bonds (Echelman et al., 2016). From one purification batch, a band at HMW was excised, trypsin digested and analyzed by mass spectrometry (LC-MALDI) (Supplementary Table 1). Six peptides matching with SpaC sequence (UniParc: UPI0001B5E4D6) were identified, confirming the presence of the pilin and therefore of pili. These peptides are found either in the CnaA or the vWFA domains (PDB: 6M48). Moreover, two peptides belonging to a protein of unknown function (matrix-binding protein, UniParc: UPI0001B5E7B8) were also identified. Surprisingly, only one peptide from SpaA (UniParc: UPI0001B5E4D4) was found in a β-sheet from one CnaB domain (PBD: 5F44). However, it did not pass the conditions confirming the presence of the protein, i.e., identifying at least two peptides having a score higher than the threshold (*p* < 0.05). This result was quite surprising, since SpaA is the major pilin and should be the most represented in the pilus. One explanation might be the resistance to trypsin digestion since the presence of the two isopeptide bonds could drastically affect digestion efficiency if they maintain a large portion of intact secondary structures, and so a resistance to SDS unfolding similarly to mechanical unfolding (Baker et al., 2015; Chaurasia et al., 2016). One peptide belonging to a murein DD-endopeptidase (UniParc: UPI0001B5E883) was also identified. Not surprisingly, no peptides from SpaB were detected since it is the least represented pilin within the pilus.

From another purification batch after MMC, it was observed that most of the CWPs were again efficiently captured by Capto Core 700 resin and only five bands were detected by SDS-PAGE: the band found at HMW, likely the pili, and four bands at 35, 50, 60, and 65 kDa (Figure 2D). In order to eliminate the low molecular weight contaminants, concentrated samples were injected from the MMC step onto a SEC column (Sephacryl HR 400, separation range for globular proteins: 20–8000 kDa). Only one peak was observed as having an elution volume (Ve) of ∼40 mL. After SDS-PAGE analysis, contaminants were still present (data not shown). Thus, it was suggested that these contaminants might co-elute with the pili, meaning that they interact with each other, or that they just have the same elution volume. To answer the question, the same protocol was used to purify CWPs from LGG Δ*spaCBA* mutant, which is devoid of pili. After CW digestion, MMC and SEC, bands at ∼35, 50, 60, and 65 kDa were still observed but no longer the band at HMW, confirming that this band corresponds effectively to the pili (data not shown). Moreover, these contaminants had the same elution volume on SEC, meaning that they do not bind pili but are certainly a complex or aggregate of HMW. To obtain more information about these proteins, they were analyzed by mass spectrometry (LC-ESI). All of them were successfully identified as ABC transporter substrate binding-related proteins (Supplementary Table 2).

To get rid of these protein contaminants and to concentrate SpaCBA pili, IEX chromatography was used. SpaA has a predicted pI of 4.7 in its mature form, i.e., without the signal peptide and without the C-term part after the LPXTG motif. Therefore, pili should bind to an anion-exchange column, while the contaminants have a pI > 9 and should not bind to the resin. Before injecting the flow-through from MMC, the ionic strength of the sample was lowered by diafiltration in Tris 25 mM pH 6.8 buffer to ensure a proper binding of the pili. The diafiltered sample was injected on strong anion exchange chromatography bearing a quaternary ammonium group. Once the sample was completely loaded and the columns washed, proteins were eluted with a linear gradient to 1M NaCl in 5 CV. Two properly separated peaks (Figure 2C) were observed, one eluting at ∼15 mS/cm and the other at 50 mS/cm (top of the peaks). The first peak contained the pili and the second one the contaminants, as shown in SDS-PAGE analysis (Figure 2D). The contaminants were not expected to remain bound to the resin since the theoretical pI is >9 and those proteins should have been positively charged. Positive charges may be hidden while negative charges are accessible in the conformation state adopted by these ABC transporter-related complex/aggregates. Although no other protein other than pili were detected on SDS-PAGE stained with Coomassie blue, UV-vis quantification showed an unusual 260 nm/280 nm ratio >1, showing that non-protein biomolecules were present with the purified pili. A simple last step of diafiltration was added to obtain highly purified pili to get rid of these contaminants.

Although the IEX steps were performed with an automatic liquid chromatography device, peaks were well separated, and operating with a syringe in step elution mode, instead of gradient, should not interfere with pili purity. Interestingly, keeping the ionic strength high after the MMC step (∼15 mS/cm) allowed the pili to flow through the column, but the contaminants were still captured. In this case, the pili were highly diluted but could easily be concentrated afterward (data not shown). Other conditions were tested during the development of this protocol including running MMC in PBS pH 7.4 with or without 300 mM NaCl, but this did not change the results. Similarly, running MMC in Tris buffer 25 mM with 150 mM NaCl did not capture more protein contaminants and a second step of IEX was mandatory to obtain purified pili. To sum up, a simple protocol based mainly on MMC that allows for the purification of SpaCBA pili from LGG was developed. In 2–3 working days, and starting from 1 L culture at OD_600nm_ ≈ 1, ∼50 μg of pili (∼2.10^13^ molecules, 500 nm long, 1.6 MDa) can be purified. OD_600nm_ ≈ 1 corresponds to a bacterial concentration of ∼1.25.10^8^ CFU/mL [considering 5 LGG per bacterial chain (Gomand et al., 2020)]. This suggests that ∼30 pili are purified per bacterium.

### Characterization of the Purified SpaCBA Pili

**FIGURE 3:**
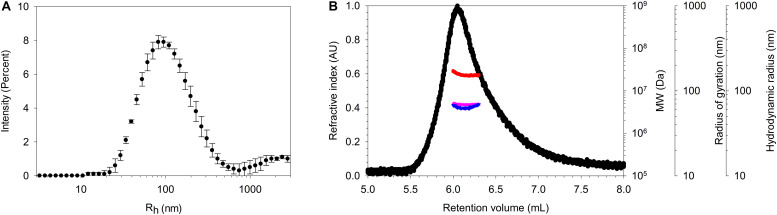
Characterization of the purified pili. **(A)** DLS analysis showing a large distribution of R_*h*_ centered on ∼ 85 nm. **(B)** SEC-TDA analysis of SpaCBA pilus giving a MW (red) at the top of the peak of ∼ 25 MDa and R_*g*_ (pink) and R_*h*_ (blue) values of 70 and 65 nm. Refractive index signal is represented in black. The void volume and the total volume of the SEC column are ∼6 and 12.5 mL respectively.

SpaCBA pili, purified thanks to this method, were characterized by DLS (Figure 3A). The mean *R*_*h*_ is centered at 82 nm and the distribution is broad since a polydispersity index (PDI) > 0.7 was obtained, as expected. Indeed, pili could harbor different hydrodynamic volumes according to the number of SpaA found in one pilus and/or they may adopt a conformation that is more or less straight or bent in solution. The purified pili were then analyzed by HPLC, more precisely by SEC coupled to multidetectors (TDA) to determine their MW, radius of gyration (*R*_*g*_) and hydrodynamic radius (*R*_*h*_) (Figure 3B). Buffer exchange was performed by diafiltration with the same buffer as the eluent to avoid buffer mismatches that could interfere with the refractometer signal at the end of the column. The first observation was that the pili eluted close to the void volume (∼6 mL) of the column designed to separate globular proteins up to 5 MDa, confirming their high MW. The MW value at the top of the peak was ∼25 MDa while *R*_*g*_ and *R*_*h*_ values were ∼70 and ∼65 nm respectively. Henceforth, the shape factor ρ (*R*_*g*_/*R_*h*_)* was approximately *1.1*, a value in line with the elongated form of the pili. From these data, it can be concluded that pili are not monomeric and might form aggregates as previously observed by Tripathi et al. (2012), who observed by AFM the formation of bundles. Differences in *R*_*h*_ could be due to a small proportion or larger pili present in DLS analysis, but not in SEC-TDA analysis, since the latter is equipped with a post-column nylon filter. Moreover, SEC-TDA data are from the top of the peak only and did not include the analysis of the whole peak.

### AFM Imaging of Dry Pili

**FIGURE 4:**
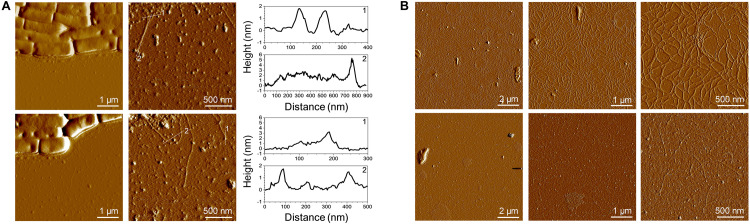
AFM imaging of SpaCBA pili. **(A)** Low and high resolution images of LGG cells presenting pili. The vertical cross sections were taken along the dashed lines on the corresponding height images. **(B)** Purified pili imaged at two concentrations (top: 50 mg/L, bottom: 2.5 mg/L).

Atomic force microscopy was employed for further characterization and imaging. Imaging pili is quite challenging and only a few publications have reported AFM imaging of these filamentous proteins. Indeed, LGG pili were successfully imaged directly on bacteria or after purification (Tripathi et al., 2012). Recently, the resolution serving to distinguish SpaA all along the pilus and SpaC at the tip was even reached (Kant et al., 2020). In the present work, pili were imaged on bacteria as well (Figure 4A). As expected, they appear to be protruding, long and thin filaments having a length around 500 nm. The height measured on a pilus on a cross-section from the base to the tip corresponds to a thickness of 2 nm all along the pilus and a value of around 4–5 nm at the top of the pilus. These values are in line with those of the aforementioned recently published work (Kant et al., 2020). Purified pili were also imaged by AFM (Figure 4B). Pili were analyzed either at 50 or at 2.5 mg/L. At the higher concentration, the pili formed a network bound and no bundles were observed as previously reported (Tripathi et al., 2012). Such bundles could have been formed due to the co-purification of EPS following the centrifugation-based protocol (Tripathi et al., 2012; Supplementary Figure 1A). Nonetheless, a thickness of ∼2 nm was measured. This value is in line with the XRC 3D structure of SpaA, the major pilin (Chaurasia et al., 2016). At this concentration and taking into account drying, this network formation might occur *via* SpaC since SpaC-SpaC interactions were highlighted by single molecule force spectroscopy with recombinant SpaC (Tripathi et al., 2013). At the lower concentration, isolated purified pili are easily detectable and, similarly to cell-attached pili, the average length was 500 nm and the thickness 2 nm (Figure 4B). No measures at the top of the purified pilus were made since the N-term C-term orientation might be tricky and could have led to mistakes.

## Conclusion

In the present study, we have developed a more versatile and easy to implement protocol than those available in the literature to purify SpaCBA pili from *Lacticaseibacillus rhamnosus* GG, one of the most widely studied probiotic bacteria, whose probiotic properties might partially occur through these pili. This novel protocol is mainly based on multimodal chromatography (core bead technology using Capto Core 700 resin), requires minimal laboratory equipment, is not expensive and can be used to obtain SpaCBA pili with a high degree of purity. Although done with a basic chromatographic system, purification might be performed with a peristaltic pump or even a simple syringe and would not require complex lab equipment nor the use of specific antibodies that are not commercially available. A custom antibody production might be expensive and only dedicated to the targeted pilins. This work could allow for the expansion of studies dedicated to SpaCBA pili at the molecular level, and the characterization of interactions with their protein partners would also be facilitated. Finally, we believe that this new purification method might be a solid basis for further purification of other sortase-dependant pili.

## Data Availability Statement

The mass spectrometry proteomics data have been deposited to the ProteomeXchange Consortium via the PRIDE (Perez-Riverol et al., 2019) partner repository with the dataset identifier PXD020875.

## Author Contributions

RDSM, JB, FB, and CG designed the study and wrote the manuscript. RDSM, BS, and SB prepared the sample. RDSM, SE-K-C, BS, and CP acquired the data. RDSM and SE-K-C analyzed or interpreted the data. RDSM drafted the manuscript. All authors reviewed the manuscript and approved the final version.

## Conflict of Interest

The authors declare that the research was conducted in the absence of any commercial or financial relationships that could be construed as a potential conflict of interest.
